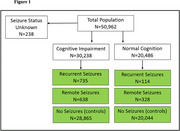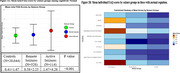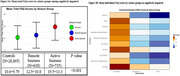# Active Seizures are Associated with Worse Instrumental Activities of Daily Living in Individuals with Normal Cognition and Dementia

**DOI:** 10.1002/alz70857_097423

**Published:** 2025-12-24

**Authors:** Ifrah Zawar, Jaideep Kapur, Meghan K Mattos, Carol A Manning, Mark S Quigg

**Affiliations:** ^1^ University of Virginia, Charlottesville, VA, USA

## Abstract

**Background:**

Cognitive deficits are common in older persons with seizures (PWS). Cognitive disorders are often characterized by impairment in Instrumental Activities of Daily Living (IADL). Despite the high occurrence of cognitive decline in older PWS, their effects on IADL remain unexplored. This study examined the associations between active versus remote seizures on the baseline IADL impairment and longitudinal decline of IADLs in older adults with and without cognitive impairment.

**Method:**

This prospective study is based on 42 US Alzheimer's Disease Research Centers from 9/2005 to 3/2024. Participants were classified into active seizures (within the past 12 months), remote seizures (prior history but none in the past 12 months), and no seizures (controls). IADLs were measured using the Pfeffer Functional Activities Questionnaire (FAQ). ANOVA compared individual and total baseline FAQ scores by seizure status and cognitive categories. Multivariable linear regression adjusted FAQ for seizure status, age, sex, and education. The rate of FAQ decline (follow‐up minus baseline score/time interval) was compared across seizure groups using ANOVA. Posthoc Tukey tests identified significant group differences.

**Result:**

Among 20486 cognitively normal participants (average age=69.7 years, female=65%[*N* = 13,370]), IADLs were worse among active seizure participants (adjusted‐mean‐difference [95% confidence interval (CI)]: active vs remote=0.69(0.23,1.14), *p* = 0.0011 and active vs controls=0.85(0.46,1.24), *p* < 0.001). Among 30,238 cognitively impaired participants (average age=72.2 years, female=52%[*N* = 15,632]), IADLs were worse among active seizure participants (adjusted‐mean‐difference (95%‐CI): active vs. remote=6.85(5.59,8.10), *p* <0.001 and active vs. controls=8.93(8.06,9.80), *p* <0.001). Longitudinally, IADLs in cognitively normal adults declined faster in those with active seizures compared to controls (*p* = 0.0396). The impairment in IADLs among active seizure participants was disproportionately higher than their level of cognitive abilities. The worst functions in active seizure participants were in the ability to assemble tax records, travel out of the neighborhood, pay bills, and, to a lesser degree, remember dates or appointments.

**Conclusion:**

Active seizures are associated with worse IADL performance regardless of cognitive status. Active seizures are also associated with a significantly faster rate of decline in IADLs in cognitively normal individuals. These findings suggest the need to assess IADLs in routine clinical care in older PWS to identify those needing assistance and for aggressive seizure management to preserve independence in at‐risk populations.